# Clinical applicability of nursing outcomes in the evolution of orthopedic patients with Impaired Physical Mobility^1^

**DOI:** 10.1590/0104-1169.3526.2524

**Published:** 2015

**Authors:** Marcos Barragan da Silva, Miriam de Abreu Almeida, Bruna Paulsen Panato, Ana Paula de Oliveira Siqueira, Mariana Palma da Silva, Letícia Reisderfer

**Affiliations:** 2 Doctoral student, Escola de Enfermagem, Universidade Federal do Rio Grande do Sul, Porto Alegre, RS, Brazil; 3 PhD, Associate Professor, Escola de Enfermagem, Universidade Federal do Rio Grande do Sul, Porto Alegre, RS, Brazil; 4 Undergraduate student in Nursing, Escola de Enfermagem, Universidade Federal do Rio Grande do Sul, Porto Alegre, RS, Brazil; 5 Master´s student, Escola de Enfermagem, Universidade Federal do Rio Grande do Sul, Porto Alegre, RS, Brazil

**Keywords:** Nursing Diagnosis, Nursing Process, Classification, Outcome Assessment (Health Care), Orthopaedic Nursing

## Abstract

**AIM:**

to evaluate the clinical applicability of outcomes, according to the Nursing Outcomes Classification (NOC) in the evolution of orthopedic patients with Impaired Physical Mobility

**METHOD:**

longitudinal study conducted in 2012 in a university hospital, with 21 patients undergoing Total Hip Arthroplasty, evaluated daily by pairs of trained data collectors. Data were collected using an instrument containing five Nursing Outcomes, 16 clinical indicators and a five point Likert scale, and statistically analyzed.

**RESULTS:**

The outcomes Body Positioning: self-initiated, Mobility, Knowledge: prescribed activity, and Fall Prevention Behavior presented significant increases in mean scores when comparing the first and final evaluations (p<0.001) and (p=0.035).

**CONCLUSION:**

the use of the NOC outcomes makes it possible to demonstrate the clinical progression of orthopedic patients with Impaired Physical Mobility, as well as its applicability in this context.

## Introduction

With longer life expectancy and the consequent increase in the number of active and independent elderly people, the surgical replacement of the hip joints are procedures increasingly used in the population with orthopedic problems^(^ 1 ^-^ 2 ^)^. The indication of Total Hip Arthroplasty (THA) should be based on the failure of conservative treatment and on the justifiable clinical condition^(^ 1 ^-^ 2 ^)^. Total Hip Arthroplasty is a widely used and effective procedure that improves the quality of life of patients by increasing functional capacity, decreasing pain and improving coxofemoral function^(^ 1 ^-^ 2 ^)^. In Brazil, this surgery was one of the most performed in the Brazilian National Health System (SUS) over the previous two years^(^ 3 ^)^. 

Much of the post-operative care, essential to the success of the surgical procedure, is the responsibility of the nurse and is directed toward the correct mobilization and education of the patient^(^ 4 ^)^. Therefore, these patients require more nursing care time, as they become dependent in the postoperative period, mainly due to mobility limitations and confinement to the bed. In spite of different interventions being carried out, the measurement of Nursing Outcomes is still new in Brazilian Nursing^(^ 5 ^)^. In this sense, to obtain desired outcomes it is necessary to establish accurate diagnoses, goals to be achieved and interventions that enable the improvement of the patient^(^ 5 ^)^.

The Nursing Outcomes Classification (NOC) was developed in order to standardize the nursing language related to the evaluation of outcomes. This classification is structured on three levels of abstraction, including Nursing Outcomes, indicators and Likert scales. The NOC aims to evaluate the progress, stagnation or worsening of the clinical condition of the patient, allowing the verification of the progress, especially as a result of the interventions prescribed and implemented by the Nurse^(^ 6 ^)^. Its interconnection with classifications used in the Diagnosis^(^ 7 ^)^ and Nursing Interventions^(^ 8 ^)^ improves clinical decision making in patient care and in monitoring the progress. 

In patients who underwent THA, the Nursing Diagnosis *Impaired Physical Mobility* ^(^ 7 ^)^ (IPM), has been highlighted as prevalent^(^ 9 ^)^. However, the clinical progression of the patient with this diagnosis, using a standardized classification, remains unexplored.

In recent years, there has been an increase in the production of studies focused on the NOC. A systematic review identified 312 articles about standardized language, with the majority of studies about the NOC focused on the reliability and validity of its terms (n=12) and the perception of nurses regarding the potential for its use in practice (n=12). However, only six studies used this classification in clinical nursing practice^(^ 10 ^)^. 

The present investigation was outlined from these considerations. The relevance of this study lies in the visibility that it can give to the clinical progression of the patient, through the use of a standardized classification. It is believed that the changes in mobility achieved by the patient may support the development of more effective care. Thus, the aim of this study was to evaluate the clinical applicability of outcomes, according to the NOC, in the progression of orthopedic patients with impaired physical mobility.

## Methods

This longitudinal study was conducted in a large university hospital in southern Brazil, accredited by the Joint Commission International. The institution has 865 beds, distributed over more than 60 specialties. The Nursing Process, used as a working method, is computerized and has the nursing diagnosis (ND) step based on the terminology of NANDA International^(^ 7 ^)^ and the prescribed care based on the Nursing Interventions Classification (NIC)^(^ 8 ^)^.

The study population consisted of patients in the THA postoperative period, hospitalized in the Surgical Nursing Service units. The sample size calculation was estimated for the improvement of the NOC score result, using the WinPepi Version 10.5 program. Considering a difference of 0.5 in the score of the results of the NOC, obtained in a pilot study, with a power of 90% and an alpha type error of 1%, it would be necessary to include 17 patients in the study, with 20% added due to possible monitoring period losses.

The consecutive type sample was selected by convenience, so that the patients were allocated in the study by admission in the units. The inclusion criteria considered were patients aged ≥18 years who underwent THA; with the IPM nursing diagnosis established by the attending nurse and recorded in the medical record; and that remained hospitalized for four days, or until discharge. This monitoring period was chosen, considering the length of hospitalization. Patients were excluded that presented clinical instability during the data collection period; were transferred to other institutions or units, or that presented limitations that prevented communication and interaction with the researchers. 

For the selection of the Nursing Outcomes, 44 results were considered, according to the NOC-NANDA-I linkage, including *suggested and additional associated* outcomes for the IPM diagnosis^(^ 6 ^)^. These outcomes were evaluated by three nurses with three or more years of clinical experience in the care of orthopedic patients. Thus, considering the title and the definition of each of the outcomes and indicators, the nurses noted the options *recommend* or *not recommend* for the evaluation of the diagnosis studied. Through the consensus, five Nursing Outcomes and 16 indicators were listed for their clinical applicability to be verified. The NOC recommendation regarding the choice of outcomes relevant to the care context in which they will be applied was taken into consideration^(^ 6 ^)^.

**Figure 1 -:**
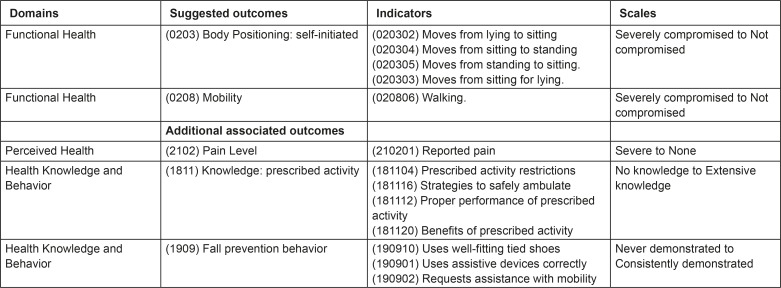
Domains, Nursing Outcomes and their indicators listed for the diagnosis of IPM in THA patients. Porto Alegre, RS, Brazil, 2013.

After this step, the data collection instrument was constructed. It contained sociodemographic and clinical variables, the five Nursing Outcomes and the 16 indicators with conceptual and operational definitions developed by the researchers from a literature review. The outcomes evaluated are presented in Figure 1.

The nurses that selected the outcomes and indicators validated the content and appearance of the instrument. Small suggestions were incorporated. The instrument was tested in a pilot study with four patients, in order to observe the variation of the indicator scores, to standardize the data collection logistics, and to support the performance of the sample calculation. It should be noted that the patients evaluated in the pilot study were not included in the final sample and the measurement scales were maintained in accordance with the NOC.

Data collection was performed by undergraduate research students that were members of a research group related to the NANDA-I nursing classifications, NIC and NOC. They underwent 18-hours of training, including theoretic lectures on the THA postoperative period and discussion of clinical cases of patients undergoing this surgery, with IPM, as well as a review of the instruments and data collection logistics.

Data were collected between August and December 2012. The logistics began with the recruitment of patients in the inpatient units. After signing the consent form, the patients were monitored with daily evaluations. Two collectors evaluated the patient simultaneously, although independently, recording the data in individual instruments. For the evaluation of the results, data from medical records, interviews and physical examinations were used, according to the conceptual and operational definitions developed for the selected clinical indicators. These indicators were evaluated by means of a five point Likert scale, where 1 corresponded to the worst score and 5 the best score, with different measurement scales of the NOC. 

The Excel 2010 software was used for the construction of the data sheets, and the Statistical Package for the Social Sciences (SPSS) version 18.0 for the data analysis.The continuous variables were expressed as mean and standard deviation for those with normal distribution or median and interquartile range for the asymmetrical variables. Categorical variables were expressed as percentages and absolute numbers. The Student's t-test for paired samples was used to compare the means between the collectors and between the first and last days of evaluation. A value of p<0.05 was considered significant. 

The study was approved by the Research Ethics Committee of the Institution, under authorization No. 110601.

## Results

The study included 21 patients, who received 68 reviews, with 15 (71.4%) of them evaluated over a four-day monitoring period, and the others over three days, according to the length of hospital stay.

Of the patients monitored, the majority were female with a mean age of 58.8 (±16.7) and 15 of them (71.4%) underwent primary THA. Osteoarthritis was the basal disease in the majority of cases, as shown in Table 1.

**Table 1 -:** Socio-demographic and clinical characteristics of the patients undergoing THA. Porto Alegre, RS, Brazil, 2013.

Variable		Total n=21
Age, years*		58.8 (±16.7)
Gender, female^†^		13 (61.9)
BMI (kg/m^2^)*		23.01 (±7.09)
Schooling, years*		8.2 (±4.1)
Caregiver presence on admission^†^		17 (80.9)
Reason for surgical indication		
	Osteoarthritis^†^	16 (76.2)
	Dislocation^†^	3 (14.2)
	Fractures^†^	2 (9.5)
Primary THA^†^		15 (71.4)
Performed preoperative outpatient nursing consultation^†^		5 (23.8)
Received preoperative nursing home visits^†^		4 (19)
Presence of pain in the hip prior to surgery^†^		20 (95.2)
Suffered a fall in the previous year^†^		10 (47.6)
Evaluation time, 4 days^†^		15 (71.4)

* Numbers expressed as mean (±standard deviation)

† n(%)

The Nursing Outcomes were measured daily, according to the clinical progression of the patients. Regarding their mean scores presented, there was a significant increase in scores in almost all evaluations, with the exception of *Pain Level* (p=0.265), as shown in Table 2.

**Table 2 -:** Mean scores of the Nursing Outcomes for patients with IPM ND undergoing THA. Porto Alegre, RS, Brazil, 2013.

Nursing Outcomes	1^st^ Day	2^nd^ Day	3^rd^ Day	4^th^ Day	p*
	Mean (± Standard Deviation)	Mean (± Standard Deviation)	Mean (± Standard Deviation)	Mean (± Standard Deviation)	
Body positioning: self-initiated	2.10 (±1.47)	3.68 (±1.20)	4.23 (±1.00)	4.48 (±0.99)	<0.001
Mobility	1.00 (±0.00)	1. 40 (±1.06)	2.93 (±1.67)	3.47 (±1.36)	<0.001
Pain level	3.87 (±0.99)	4.00 (±0.93)	4.27 (±0.96)	4.20 (±1.27)	0.265
Knowledge: prescribed activity	3.12 (±0.51)	3.52 (±0.55)	3.75 (±0.39)	3.64 (±0.56)	0.035
Fall prevention behavior	2.15 (±0.72)	2.78 (±0.77)	3.29 (±0.75)	3.55 (±0.56)	<0.001

* Paired student's t-test

**Figure 2 -:**
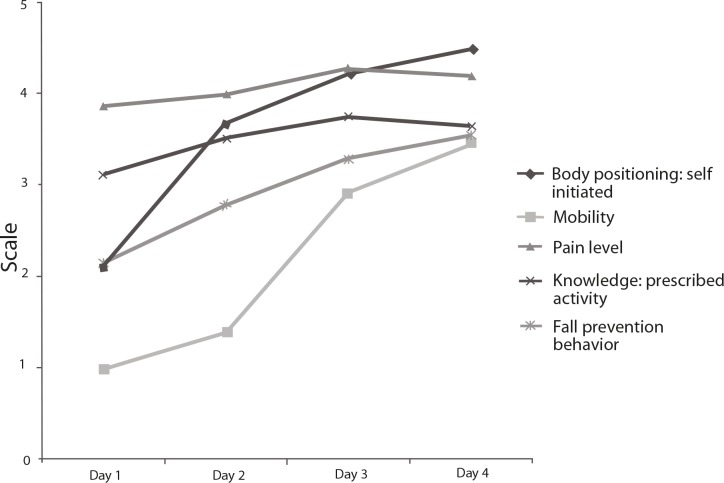
Temporal curves for the Nursing Outcomes in patients with IPM undergoing THA. Porto Alegre, RS, Brazil, 2013.

Regarding the clinical evolution, the temporal curves show the differences in the scores of the scales of the NOC outcomes for each day evaluated. As can be seen in Figure 2, an increase was verified in virtually all evaluations.

In the comparison between the mean scores of the first and last evaluations of the patients, no significant difference was observed in the measurements of the collectors. The mean difference was no more than 0.35 points in any of the parameters analyzed, as shown in Table 3.

**Table 3 -:** Comparison of the mean scores of the NOC Nursing Outcomes between the evaluators of the patients with IPM ND undergoing THA. Porto Alegre, RS, Brazil, 2013.

Outcomes	N	Evaluator 1	Evaluator 2	Difference (CI 95%)	p*
		Mean (± Standard Deviation)	Mean (± Standard Deviation)		
Body positioning: self-initiated	2115	1.79 (±1.33)4.48 (±0.99)	1.81 (±1.38)4.38 (±0.96)	0.02 (-0.02 - 0.07)0.10 (-0.01 - 0.21)	0.3290.082
					
Mobility	2115	1.00 (±0.00)3.47 (±1.36)	1.00 (±0.00)3.40 (±1.35)	0.00 (0.00 - 0.00)0.07 (-0.08 - 0.21)	1.0000.334
					
Pain level	2115	3.76 (±1.22)4.20 (±1.27)	3.76 (±1.22)4.13 (±1.06)	0.00 (0.00 - 0.00)0.07 (-0.19 - 0.32)	1.0000.582
					
Knowledge: prescribed activity	2115	3.12 (±0.53)3.64 (±0.56)	3.05 (±0.65)3.57 (±0.50)	0.07 (-0.06 - 0.21)0.07 (-0.16 - 0.31)	0.2840.515
					
Fall prevention behavior	2115	2.28 (±0.69)3.55 (±0.56)	2.41 (±0.89)3.58 (±0.61)	0.13 (-0.08 - 0.33)0.02 (-0.15 - 0.20)	0.2100.788

* Paired student's t-test

## Discussion

This study monitored 21 patients with IPM in the THA postoperative period, aiming to verify the clinical applicability of five Nursing Outcomes contained in the Functional Health, Perceived Health, and Health Knowledge and Behavior domains^(^ 6 ^)^. It should be noted that the study did not aim to evaluate the validity of the NOC scales, but the applicability of this classification in clinical practice, observing the changes of the health status of patients with the progression of the nursing care.

Regarding the Body Positioning: self-initiated Outcome, this showed a progressive increase in the mean scores of the indicators evaluated in the daily monitoring of the patients. It should be noted that the mean scores identified by the examiners did not present statistically significant differences, demonstrating consistency in the evaluations. For patients who have undergone THA it is recommended that the legs are kept abducted and with hip flexion greater than 90º, in order to prevent displacement of the prosthesis^(^ 11 ^)^. They are informed about the need for correct positioning in all the movements performed. These guidelines are contained in a clinical protocol and support nurses in the safe handling of these patients, facilitating the management of the positioning during care activities^(^ 12 ^)^.

The clinical improvements in the Body Positioning: self-initiated Outcome and the Mobility Outcome, which also presented a significant improvement in the mean scores (p<0.001), were also found the literature, as mobility is a term that has been used to explain a series of functional activities, including transfer from the bed to the chair and walking^(^ 13 ^)^. The operational definitions of these outcomes included, for example, whether the patient maintained the proper positioning to sit on the bed, or when transferring from the bed to a chair, or vice versa; whether the patient started the first step with the operated limb; whether they kept the leg straight, distributed the weight with crutches or a walking frame; and important nursing care to be evaluated during the movement of these patients^(^ 11 ^)^. In addition to these evaluations, the degree of impairment of the Nursing Diagnosis under study can be perceived by measuring the Nursing Outcomes, which gradually improved over the days of monitoring.

It was observed that the study sample presented a mean body mass index (BMI) of 23.01 kg/m^2^ (±7.09), which indicates normal weight. This data may have influenced the improvement of Mobility, supported by the research findings that showed the BMI to be a predictor for the outcomes of the THA^(^ 14 ^)^.

The Pain Level Nursing Outcome (p=0.265) showed no statistically significant difference, with the mean scores between the collectors being excellent in the first evaluation and, in the last, the variation was only 0.07 in the Reported Pain indicator scores. In the study setting, pain is evaluated as the fifth vital sign; thus, it is believed that the data can be related to the greater attention given to patients with the possibility of acute pain^(^ 15 ^)^.

Pain is a subjective phenomenon of extremely complex perception. As a factor related to the IPM diagnosis, patients who will have a hip prosthesis fitted learn to live with pain in their daily activities and not seek help until it becomes unbearable^(^ 16 ^)^. The algic perception was present in 20 (95.2%) patients prior to the surgery. Accordingly, it can be inferred that the pain was higher in the period leading up to the surgery, when compared to the postoperative period^(^ 16 ^)^.

Regarding the Knowledge: prescribed activity Outcome, categorized as additional associated in the NOC-NANDA-I linkage for the diagnosis in question, its evolution was statistically significant in this study (p=0.035). It is believed that the educational activities performed preoperatively contributed to the patients presenting moderate knowledge regarding the activities that they may or may not perform in the postoperative period, represented by the score of 3 in the NOC scale. In addition, in the institution under study, patients undergoing THA receive a manual from the nursing team with guidelines on the care needed after discharge^(^ 11 ^)^. This resource assists in the compression of the surgery and in the care that should be performed at home. Researchers emphasize that combined clinical and educational interventions can help patients awaiting surgery^(^ 17 ^)^. 

The Fall prevention behavior Nursing Outcome presented progressive improvement in the mean scores (p<0.001) for the patients monitored. This result was applicable in clinical practice, considering that patient safety is the focus of the nursing care in this hospital. In addition, 17 (80.9%) of the study patients were monitored by caregivers during the hospitalization, a factor that may have helped in fall prevention behavior^(^ 18 ^)^.

Although none of the patients in this study suffered falls during the monitoring, the related literature highlights the need to establish evaluation and fall prevention programs after arthroplasty surgeries. This policy becomes imperative due to the risks presented by patients undergoing THA, who mostly have functional limitations and advanced age^(^ 19 ^)^. In the study sample, the NOC scores showed that the patients presented from moderate to frequent fall prevention behavior, demonstrated throughout the evaluations. Accordingly, it is understood that this preventive behavior is inherent to individuals, who assume a positive attitude towards their health, in order to reduce their susceptibility, avoid the subsequent emergence of diseases and thus preserve their integrity^(^ 20 ^)^.

Regarding the mean scores between the collectors, there were no statistically significant differences in the evaluations for any of the NOs. A study that compared the interobserver concordance of patients evaluated with the use of operational definitions for the clinical indicators of the Ineffective Breathing Patterns ND verified inconsistencies in the evaluations of those who did not use them^(^ 21 ^)^.

Thus, the monitoring of the progress of patients, through a standardized classification, can facilitate evidence-based practice, favoring the quality of care and comprehensiveness of documentation, through the use of internationally recognized nursing language systems, which are valid and applicable in different real clinical settings^(^ 22 ^-^ 25 ^)^.

## Conclusion

The use of the NOC outcomes made it possible to demonstrate the clinical progression of orthopedic patients with Impaired Physical Mobility, as well and its applicability in this context. It was possible to observe the status of the diagnosis under study, from the scores of the outcomes contained in the functional health, perceived health, and patient knowledge domains on each evaluated day. In addition, through the comparison of the mean scores between the collectors, the consistency of the evaluations, using an instrument constructed for this purpose, could be seen. However, the small sample size, the coverage of the NOC and the possibility of choice of the outcomes for different populations hindered the validation using psychometric criteria common in scale validation studies, which limits the generalizability of these findings.

As implications for practice, the construction and validation of conceptual and operational definitions for specific contexts is suggested, as well as training for nursing teams. The development of these activities prior to the implementation of the NOC, may facilitate its use in clinical practice and encourage the evaluation of the effectiveness of interventions, through the monitoring of nursing care outcomes. The measurement of the time of evaluation of these results can maximize the impact of the applicability of these findings.

More studies on this topic are needed to establish the validity of the classification and of the possible comparisons with other populations and practice contexts.
